# The senolytic agent ABT263 ameliorates osteoporosis caused by active vitamin D insufficiency through selective clearance of senescent skeletal cells

**DOI:** 10.1016/j.jot.2024.08.012

**Published:** 2024-10-05

**Authors:** Cuicui Yang, Wanxin Qiao, Qi Xue, David Goltzman, Dengshun Miao, Zhan Dong

**Affiliations:** a The Research Center for Bone and Stem Cells, Department of Anatomy, Histology and Embryology, Nanjing Medical University, Nanjing, China; b Calcium Research Laboratory, McGill University Health Centre and Department of Medicine, McGill University, Montreal, Quebec, H4A 3J1, Canada; c Department of Orthopedics, Children's Hospital of Nanjing Medical University, Nanjing, China

**Keywords:** Cellular senescence, Osteoporosis, SASP, Senolytics, Vitamin D insufficiency

## Abstract

**Background/Objective:**

Active vitamin D insufficiency accelerates the development of osteoporosis, with senescent bone cells and the senescence-associated secretory phenotype (SASP) playing crucial roles. This study aimed to investigate whether the senolytic agent ABT263 could correct osteoporosis caused by active vitamin D insufficiency by selectively clearing senescent cells.

**Methods:**

Bone marrow mesenchymal stem cells (BM-MSCs) from young and aged mice were treated with ABT263 in vitro, and 1,25(OH)_2_D-insufficient (Cyp27b1^+/−^) mice were administered ABT263 in vivo. Cellular, molecular, imaging, and histopathological analyses were performed to compare treated cells and mice with control groups.

**Results:**

ABT263 induced apoptosis in senescent BM-MSCs by downregulating Bcl2 and upregulating Bax expression. It also induced apoptosis in senescent BM-MSCs from 1,25(OH)_2_D-insufficient mice. ABT263 administration corrected bone loss caused by 1,25(OH)_2_D insufficiency by increasing bone density, bone volume, trabecular number, trabecular thickness, and collagen synthesis. It also enhanced osteoblastic bone formation and reduced osteoclastic bone resorption in vivo. ABT263 treatment corrected the impaired osteogenic action of BM-MSCs by promoting their proliferation and osteogenic differentiation. Furthermore, it corrected oxidative stress and DNA damage caused by 1,25(OH)_2_D insufficiency by increasing SOD-2 and decreasing γ-H2A.X expression. Finally, ABT263 corrected bone cell senescence and SASP caused by 1,25(OH)_2_D insufficiency by reducing the expression of senescence and SASP-related genes and proteins.

**Conclusion:**

ABT263 can correct osteoporosis caused by active vitamin D insufficiency by selectively clearing senescent skeletal cells, reducing oxidative stress, DNA damage, and SASP, and promoting bone formation while inhibiting bone resorption. These findings provide new insights into the potential therapeutic application of senolytic agents in the treatment of osteoporosis associated with active vitamin D insufficiency.

**The translational potential of this article:**

This study highlights the therapeutic potential of ABT263, a senolytic compound, in treating osteoporosis caused by active vitamin D insufficiency. By selectively eliminating senescent bone cells and their associated SASP, ABT263 intervention demonstrates the ability to restore bone homeostasis, prevent further bone loss, and promote bone formation. These findings contribute to the growing body of research supporting the use of senolytic therapies for the prevention and treatment of age-related bone disorders. The translational potential of this study lies in the development of novel therapeutic strategies targeting cellular senescence to combat osteoporosis, particularly in cases where vitamin D insufficiency is a contributing factor. Further clinical studies are warranted to validate the efficacy and safety of ABT263 and other senolytic agents in the treatment of osteoporosis in humans.

## Graphical abstract

**Figure:**
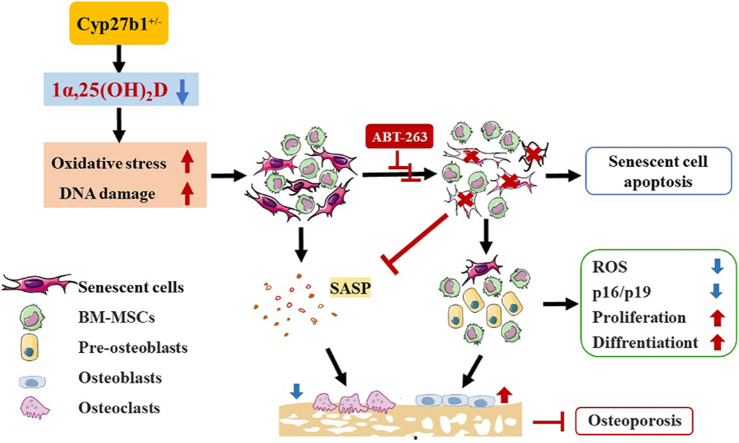

## 1 Introduction

Osteoporosis, characterized by low bone mass and increased fracture risk, is a significant global health concern, particularly in aging populations. Vitamin D insufficiency has been identified as a major contributing factor to the development of osteoporosis, as it accelerates the loss of bone mass and strength [1,2]. Previous studies have shown that an insufficiency in the biologically active form of vitamin D, 1,25-dihydroxyvitamin D (1,25(OH)₂D), can induce cellular senescence and the senescence-associated secretory phenotype (SASP) in bone cells, including osteoblasts, osteocytes, and bone marrow mesenchymal stem cells (BM-MSCs) [[3], [4], [5]]. This cellular senescence and SASP contribute to the disruption of bone homeostasis, leading to an imbalance between bone formation and resorption, ultimately resulting in accelerated bone loss and osteoporosis development.

Current therapies for vitamin D insufficiency-induced osteoporosis primarily include vitamin D supplementation and conventional osteoporosis treatments such as bisphosphonates, hormone replacement therapy, RANKL inhibitors, and anabolic agents. However, these approaches have significant limitations. Vitamin D supplementation can have inconsistent absorption rates and potential toxicity at high doses [6,7]. Bisphosphonates may cause serious side effects and have reduced efficacy in vitamin D deficient patients [8]. Hormone replacement therapy carries risks of breast cancer and cardiovascular events [9]. RANKL inhibitors can lead to rapid bone loss upon discontinuation [10], while anabolic agents are costly and have usage restrictions due to potential cancer risks [11]. These limitations, coupled with the complex nature of vitamin D insufficiency-induced osteoporosis, underscore the need for new therapeutic approaches. Novel strategies targeting underlying mechanisms, such as cellular senescence, could potentially offer more effective and targeted treatments with fewer side effects [12].

Cellular senescence, a state of irreversible cell cycle arrest, has been recognized as a key driver of age-related pathologies, including osteoporosis [13]. Senescent cells accumulate in various tissues with aging, secreting a range of pro-inflammatory cytokines, chemokines, and matrix-degrading enzymes, collectively known as the SASP [14]. In the context of bone metabolism, the SASP can promote osteoclastogenesis and inhibit osteoblastogenesis, leading to increased bone resorption and decreased bone formation, respectively [15]. Furthermore, senescent cells exhibit diminished proliferative capacity and impaired differentiation potential, which can compromise the regenerative capacity of bone-forming precursor cells, such as BM-MSCs [16].

To combat the detrimental effects of cellular senescence on bone health, senolytic therapies have emerged as a promising approach [17]. Senolytics are a class of compounds that selectively induce apoptosis in senescent cells while sparing non-senescent cells, thereby reducing the burden of senescent cells and their associated SASP [18]. Several studies have demonstrated the efficacy of senolytics in preventing and treating osteoporosis [13,19]. ABT263 (Navitoclax), a potent inhibitor of the anti-apoptotic proteins Bcl-2 and Bcl-xL, has been extensively studied as a senolytic agent [20]. Previous research has shown that ABT263 can effectively eliminate senescent cells in various tissues, including bone, by inducing apoptosis through the downregulation of Bcl-2 and the upregulation of pro-apoptotic proteins, such as Bax [18,21]. Importantly, ABT263 has been found to selectively target senescent cells while sparing non-senescent cells, reducing the risk of adverse effects associated with non-selective cytotoxicity [22].

In this study, we aimed to investigate the potential of ABT263 as a senolytic intervention to induce apoptosis in senescent BM-MSCs and correct the accelerated bone loss caused by 1,25(OH)_2_D insufficiency. By leveraging the senolytic activity of ABT263, we sought to eliminate the detrimental effects of senescent cells on bone homeostasis and potentially restore the impaired osteogenic capacity of BM-MSCs in the context of 1,25(OH)_2_D insufficiency.

The results presented in this study provide compelling evidence for the therapeutic potential of ABT263 in counteracting osteoporosis induced by 1,25(OH)₂D insufficiency. Thus, ABT263 is shown to mitigate oxidative stress, DNA damage, cellular senescence, and SASP in bone cells, and restore the impaired osteogenic differentiation of BM-MSCs caused by 1,25(OH)₂D insufficiency. The findings thus demonstrate that ABT263 intervention can correct bone loss by promoting osteoblastic bone formation, enhancing collagen synthesis and reducing osteoclastic bone resorption. These findings contribute to a growing body of research supporting the use of senolytic therapies, such as ABT263, for the prevention and treatment of osteoporosis, particularly in cases where vitamin D insufficiency is a contributing factor. By selectively eliminating senescent cells and alleviating the associated SASP, senolytics like ABT263 have the potential to restore bone homeostasis and prevent further bone loss, offering a promising therapeutic approach for this debilitating condition.

## 2 Materials and methods

### 2.1 Animal experiments

Cyp27b1^+/−^ mice were generated through the breeding of heterozygous mice and were genotyped as previously reported [3,23]. All mice were maintained on a C57BL/6J background. The mice were housed in a pathogen-free facility, subjected to a 12-h light/dark cycle, and provided with ad libitum access to food and water. To investigate the effects of ABT263 intervention, an experimental study was conducted using 11-month-old wild-type (WT) and Cyp27b1^+/−^ mice. The WT male mice received a vehicle, while the littermate Cyp27b1^+/−^ male mice were administered either a vehicle or ABT263. The administration of ABT263 was done orally by gavage, with a dose of 50 mg per kilogram of body weight per day (mg/kg/day), given for one week [21]. This was followed by a two-week interval, and then repeated for two cycles. Twelve-month-old male vehicle-treated WT and Cyp27b1^+/−^, and ABT263-treated Cyp27b1^+/−^ littermates were included in this study. The use of all mice was approved by the Institutional Animal Care and Use Committee of Nanjing Medical University (Approval number: IACUC-1802007), and they were maintained in the SPF Laboratory Animal Center of Nanjing Medical University.

### 2.2 Micro-computed tomography (micro-CT)

The vertebral columns of each mouse from L1 to L4 were isolated from the spine and soft-tissue was removed to reduce muscles and ligament interference. A micro-CT scanner (Skyscan, 1176) was used to evaluate the spine samples as described previously [24]. The scans were performed using the following scanner settings: voltage 45 kV, current 500 μA and a 780 ms integration time. The resolution of the computed image is 2672 x 4000 and the slice thickness is 9 μm. The cross-sectional images were reconstructed to three-dimensional diagrams and analyzed using a CTAnalyzer (SkyScan-10) analysis program. For trabecular bone analysis, the region of interest (ROI) started at 0.5 mm proximal to the growth plate and extended 1.5 mm in the proximal direction. Image reconstruction was performed using NRecon software (version 1.7.3.0, Bruker) with beam hardening correction set to 30 % and ring artifact correction set to 8. The reconstructed images were analyzed using CTAn software (version 1.18.4.0, Bruker). For trabecular bone analysis, the following parameters were quantified: bone volume fraction (BV/TV), trabecular thickness (Tb.Th), trabecular number (Tb.N), and trabecular separation (Tb.Sp). For cortical bone analysis, we measured cortical thickness (Ct.Th) and cortical area (Ct.Ar). The analysis threshold was determined using the automated thresholding function in CTAn, which uses the Otsu method to separate bone from background. The threshold values were then visually verified and adjusted if necessary to ensure accurate representation of the bone structure. The same threshold was applied to all samples within each experimental group to ensure consistency.

### 2.3 Histology and histochemistry

Vertebral samples were isolated and histologically processed as previously described [4]. Paraffin sections were stained for hematoxylin and eosin (H&E), and for total collagen, histochemically for ALP and TRAP as previously described [4,25].

### 2.4 Immunohistochemical staining

Immunohistochemical staining was conducted using the avidin-biotin-peroxidase complex technique, as previously described [4]. The sections of paraffin-embedded tissues were first dewaxed and rehydrated. To block the endogenous peroxidase activity, the sections were then incubated with 6 % hydrogen peroxide, followed by a wash with PBS (pH 7.6). Subsequently, the slides were incubated overnight at 4 °C with primary antibodies against SOD2 (NB100-1992, Novus Biologicals, Colorado, USA), γ-H2A.X (Ser139) (#80312S, Cell Signaling Technology, Beverly, MA, USA), β-gal (ab616, Abcam), p16 (Santa Cruz), p21 (Santa Cruz), p53 (Cell Signaling Technology), and IL-1β (ab9722, Abcam). After a 15-min rinse with PBS, the tissues were incubated with secondary antibodies (biotinylated goat anti-rabbit IgG and goat anti-mouse IgG, Sigma). Following another wash, the sections were incubated with the Vectastain Elite ABC reagent (Vector Laboratories) for 30 min. Staining was achieved using 3,3-diaminobenzidine (2.5 mg/ml) and counterstaining was performed with Mayer's hematoxylin.

### 2.5 Computer-assisted image analysis

After H&E staining or histochemical or immunohistochemical staining of sections from six mice of each group, images of fields were photographed. Images of micrographs from single sections were digitally recorded using a rectangular template, and recordings were processed and analyzed using ImageJ software as described [26,27]. For histological and immunohistochemical analyses, we examined 5 random fields per section at 200× magnification. This was consistent across all staining methods (H&E, ALP, TRAP, and immunohistochemical stains). We analyzed 3 non-consecutive levels per vertebra, spaced approximately 50 μm apart, to ensure a representative sampling of the tissue. All histological and immunohistochemical analyses were performed by two independent observers who were blinded to the experimental groups. The results of observers were then averaged for each sample.

### 2.6 Cell cultures

Tibiae and femurs were removed under aseptic conditions, and bone marrow cells were flushed out with the cell culture medium. Cells were dispersed by repeated pipetting, and a single cell suspension was achieved by forcefully expelling the cells through a 22-gauge syringe needle. Isolated cells were cultured in α-modified Eagle's MEM (Life Technologies) supplemented with 10 % FBS (Excell) and 1 % penicillin/streptomycin (Life Technologies) at a cell density of 10 × 10^6^/ml as previously described [28]. On day 3, nonadherent cells were removed using medium change and fresh medium was replaced twice per week. For osteogenic differentiation, cells were maintained in osteogenic medium (supplemented with 100 nM dexamethasone, 10 mM β-glycerophosphate, and 50 μg/ml ascorbic acid) for 10 days. Staining with Methylene blue for colony forming units-fibroblast (CFU-f) and alkaline phosphatase (Alp)-positive colonies (CFU-fap) was performed. SA-β-gal staining and EdU assay were performed as previously described [5]. ImageJ software was used to determine areas of staining.

### 2.7 Cell viability assay

Cell viability was assessed using the MTT (3-(4,5-dimethylthiazol-2-yl)-2,5-diphenyltetrazolium bromide) assay. BM-MSCs from young (2-month-old) and aged (18-month-old) mice were seeded in 96-well plates and treated with various concentrations of ABT263 (0–10 nM) for 24 h. MTT solution was then added to each well and incubated for 4 h at 37 °C. The formazan crystals were dissolved in DMSO, and the absorbance was measured at 570 nm using a microplate reader.

### 2.8 Apoptosis assay

Apoptosis was evaluated using flow cytometry with Annexin V-FITC and propidium iodide (PI) staining. BM-MSCs from aged mice were treated with 10 nM ABT263 for 48 h. Cells were then harvested, washed with PBS, and stained with Annexin V-FITC and PI according to the manufacturer's instructions. The percentage of apoptotic cells was determined using a FACS caliber flow cytometer (Becton Dickinson, Heidelberg, Germany).

### 2.9 TUNEL and p16 immunofluorescence staining

TUNEL staining was executed according to the manufacturer's instructions (Roche). Cells were counterstained with DAPI (Sigma) for 3 min. For dual staining of TUNEL and p16, following TUNEL staining, cells were rinsed twice with PBS. The primary antibody against p16 (SC-1661), diluted 1:400 in PBS, was applied and incubated overnight. After a brief incubation at room temperature, cells were washed thrice with 0.1 % PBST and incubated with the secondary antibody in darkness. Samples were re-stained with a freshly prepared 2 μg/ml DAPI solution in PBS, left at room temperature for 5 min. Slides were washed thrice by immersion in PBS and mounted with 100 μl of 20 % glycerol-PBS solution to maintain sample hydration. Positive cell percentages were quantified using ImageJ software.

### 2.10 Real-time RT-PCR

Total RNA was extracted from the cultured BM-MSCs and vertebrae using Trizol reagent (Invitrogen) according to the manufacturer's instructions. Complementary DNA (cDNA) was synthesized using Synthesis SuperMix (Invitrogen). Real-time RT-PCR was carried out using an Agilent Real-time System. Gapdh was amplified at the same time to normalize gene expression. Each experiment was repeated three times to determine relative gene expression differences. The sequence-specific primers of human and mice are displayed in Table 1.

**Table 1:** Primers used in this study for real time RT-PCR.

Name	Sence sequence	Anti-Sence sequence
BCL-2	GACAAGGAGATGCAGGTATTGG	TCCCGTAGAGATCCACAAAAGT
BAX	TGAAGACAGGGGCCTTTTTG	AATTCGCCGGAGACACTCG
RANKL	CAGCATCGCTCTGTTCCTGTA	CTGCGTTTTCATGGAGTCTCA
OPG	ACCCAGAAACTGGTCATCAGC	ACCCAGAAACTGGTCATCAGC
Osterix	CCCTTCTCAAGCACCAATGG	AAGGGTGGGTAGTCATTTGCATA
OCN	GCTGCCCTAAAGCCAAACTCT	AGAGGACAGGGAGGATCAAGTC
Runx2	GTGACACCGTGTCAGCAAAG	GGAGCACAGGAAGTTGGGAC
p16	CGCAGGTTCTTGGTCACTGT	TGTTCACGAAAGCCAGAGCG
p21	CCTGGTGATGTCCGACCTG	CCATGAGCGCATCGCAATC
p53	GCGTAAACGCTTCGAGATGTT	TTTTTATGGCGGGAAGTAGACTG
IL-1β	GCAACTGTTCCTGAACTCAACT	ATCTTTTGGGGTCCGTCAACT
IL-8	GCTGTGACCCTCTCTGTGAAG	CAAACTCCATCTTGTTGTGTC
MMP3	ACATGGAGACTTTGTCCCTTTTG	TTGGCTGAGTGGTAGAGTCCC
MMP13	CTTCTTCTTGTTGAGCTGGACTC	CTGTGGAGGTCACTGTAGACT
GAPDH	TGGATTTGGACGCATTGGTC	TGGATTTGGACGCATTGGTC

### 2.11 Western blotting

Western blotting was performed in this study to analyze protein expression levels of various target molecules. Whole cell lysates were prepared using RIPA buffer. The protein samples were then separated by SDS-PAGE and transferred onto PVDF membranes. Subsequently, the membranes were probed with specific primary antibodies against the following proteins: BCL2 (Abcam), Bax (Santa Cruz), Runx2 (Santa Cruz), Osteocalcin (Santa Cruz), SOD2 (NB100-1992, Novus Biologicals), γ-H2A.X (#80312S, Cell Signaling Technology), p16 (Santa Cruz), TNFα (Santa Cruz), and β-actin (Cell Signaling Technology). Immunoblotting was performed using HRP-conjugated secondary antibodies. The immunoreactive bands were detected using enhanced chemiluminescence (ECL) (Bio-Rad) and subsequently analyzed using ImageJ software.

### 2.12 Intracellular ROS analysis

To analyze the intracellular ROS levels, bone marrow cells of 12-month-old male vehicle-treated WT and Cyp27b1^+/−^, and ABT263-treated Cyp27b1^+/−^ littermates were flushed out from femurs and labeled with 5 mM DCFDA (diacetyldichloroﬂuorescein) in the dark for 30 min in a water bath at 37 °C, then detected with the FACS caliber ﬂow cytometer (Becton Dickinson, Heidelberg, Germany).

### 2.13 Statistics

Data are presented as mean ± SEM from at least three independent experiments. Differences between two groups were analyzed by two-tailed Student's t-test, and multiple groups by one-way ANOVA with post-hoc tests using GraphPad Prism 8. P < 0.05 was considered statistically significant. For our primary outcome measure (bone mineral density), we assumed an effect size of 1.5 (based on our preliminary data), an alpha level of 0.05, and a desired power of 0.8. The power analysis indicated that a minimum of 6 mice per group would be required to detect statistically significant differences between the groups.

## 3 Results

### 3.1 ABT263 induces apoptosis in senescent BM-MSCs accompanied by downregulating Bcl2 and upregulating Bax expression levels

**Figure 1:**
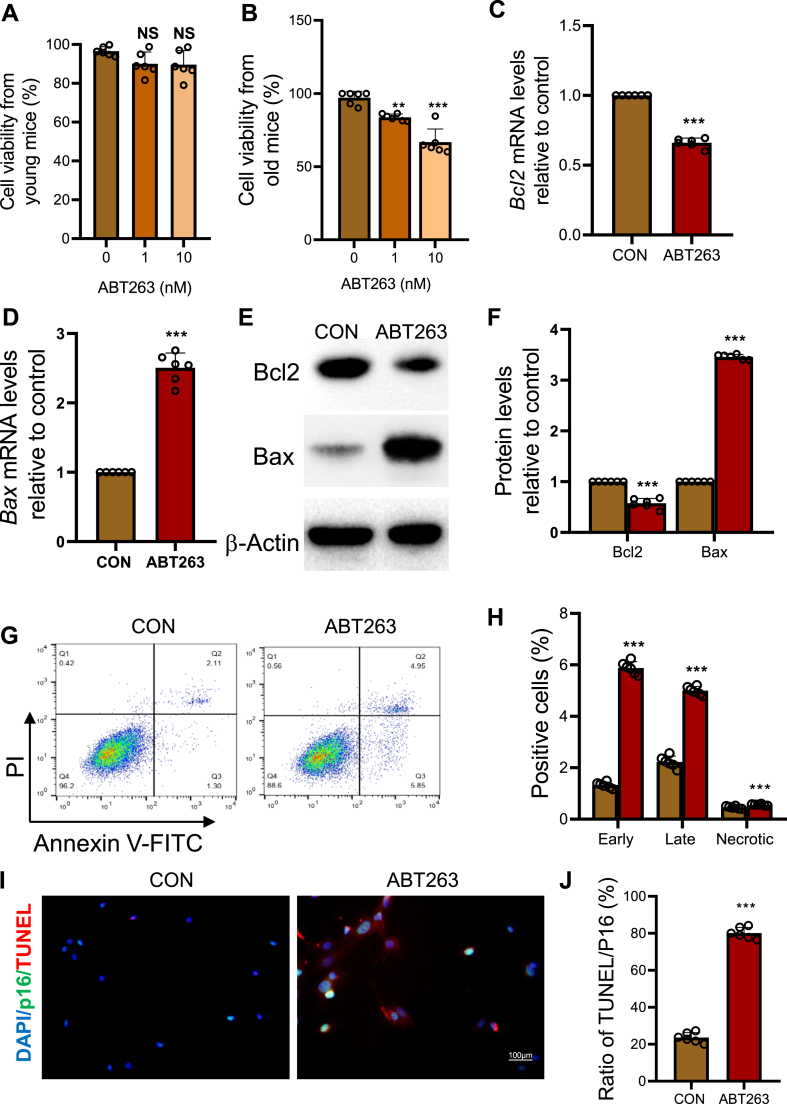
ABT263 induces apoptosis in senescent BM-MSCs by downregulating Bcl2 and upregulating Bax expression levels. Viability of BM-MSCs derived from (A) young mice (2M) and (B) aged mice (18M) treated with ABT263 at different concentrations. qRT-PCR results of relative expression levels of (C) *Bcl2* mRNA and (D) pro-apoptotic gene *Bax* mRNA. (E) Western blot results of relative expression levels of Bcl2 and Bax proteins. (F) Bar graph showing the relative expression levels of Bcl2 and Bax proteins. (G) Representative flow cytometry results showing apoptotic cells in ABT263-treated BM-MSCs derived from aged mice. (H) Bar graph showing the percentages of early and late apoptotic cells and necrotic cells. (I) Representative micrographs displaying double fluorescence staining for p16 and TUNEL. (J) The ratio of TUNEL-positive cells to p16-positive cells. Values are mean ± S.E.M. of 6 determinations per group. ∗∗: P < 0.01; ∗∗∗: P < 0.001, compared to the control group.

To determine the efficacy of ABT263 in eliminating senescent BM-MSCs, we treated BM-MSCs derived from 2-month-old young mice and 18-month-old aged mice with various concentrations of ABT263. After a 24-h incubation period, we observed that ABT263 at concentrations ranging from 1 to 10 nM had no impact on the viability of BM-MSCs from young mice. However, it substantially decreased the viability of BM-MSCs from aged mice (Fig. 1A and B).

To investigate the molecular mechanism behind this effect, we treated BM-MSCs from aged mice with 10 nM ABT263 for 48 h. Subsequently, the expression levels of mRNA and protein, the proportion of early and late apoptotic cells, and the ratio of apoptotic to senescent cells were analyzed. The results revealed a significant downregulation of mRNA and protein expression levels of Bcl2, an anti-apoptotic molecule (Fig. 1C–E & F). In contrast, the mRNA and protein expression levels of Bax, a pro-apoptotic molecule, the percentage of early and late apoptotic cells and necrotic cells and the ratio of TUNEL-positive cells to p16-positive cells were significantly increased in the ABT263-treated group compared to the control group (Fig. 1D–J). These findings demonstrate that ABT263 treatment induces apoptosis in senescent BM-MSCs, and this is accompanied by downregulation of Bcl2 and upregulation of Bax expression levels.

### 3.2 ABT263 induces apoptosis in senescent BM-MSCs from 1,25(OH)_2_D-Insufficient mice

**Figure 2:**
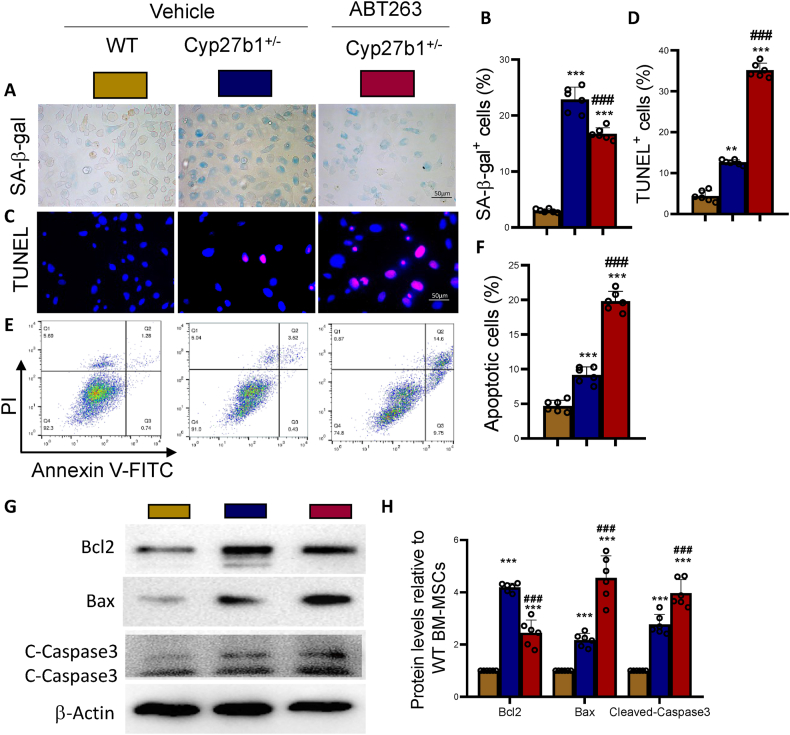
ABT263 induces apoptosis in senescent BM-MSCs from 1,25(OH)_2_D-insufficient mice BM-MSCs were isolated and cultured BM-MSCs from WT and Cyp27b1^+/−^ mice with vehicle or from the Cyp27b1^+/−^ mice with 10 nM ABT263 for 24 h. (A) Representative micrographs of SA-β-gal staining. (B) Percentage of SA-β-gal positive cells. (C) Representative micrographs of TUNEL staining. (D) Percentage of TUNEL-positive cells. (E) Representative flow cytometry results showing apoptotic cells. (F) Percentage of apoptotic cells. (G) Western blot detection of Bcl2, Bax, and cleaved-Caspase3 (C- Caspase3) proteins in thoracic vertebrae tissues. (H) Relative expression levels of Bcl2, Bax, and cleaved-Caspase3 proteins. Values are mean ± S.E.M. of 6 determinations per group. ∗∗∗: P < 0.001, compared to WT mice. ##: P < 0.01; ###: P < 0.001, compared to Cyp27b1^+/−^ BM-MSCs.

To examine the ability of ABT263 to induce apoptosis in senescent bone marrow-derived mesenchymal stem cells (BM-MSCs) obtained from 1,25(OH)_2_D-insufficient mice, we isolated and cultured BM-MSCs from 11-month-old wild-type (WT) mice and Cyp27b1^+/−^ mice. Additionally, we treated BM-MSCs from Cyp27b1^+/−^ mice with 10 nM ABT263. After 24 h, we assessed cellular senescence, apoptosis, and the expression levels of associated molecular markers in WT mice, Cyp27b1^+/−^ mice, and ABT263-treated Cyp27b1^+/−^ mice-derived BM-MSCs. The results revealed that the percentage of senescence-associated β-galactosidase (SA-β-gal) positive senescent and TUNEL-positive apoptotic cells was higher in BM-MSCs derived from Cyp27b1^+/−^ mice-compared to BM-MSCs derived from WT mice (Fig. 2A–F). Interestingly, the protein expression levels of both the anti-apoptotic gene, Bcl2, and the pro-apoptotic genes, Bax and cleaved-Caspase3, were significantly elevated in BM-MSCs derived from Cyp27b1^+/−^ mice (Fig. 2G and H), suggesting a complex dysregulation of apoptotic pathways. However, following ABT263 treatment, the percentage of SA-β-gal-positive senescent cells decreased significantly, while the percentage of TUNEL-positive apoptotic cells increased further (Fig. 2A–F). Additionally, the expression levels of Bcl2 protein decreased significantly, whereas the expression levels of Bax and cleaved-Caspase3 protein increased significantly in ABT263-treated BM-MSCs derived from Cyp27b1^+/−^ mice (Fig. 2G and H). Thus, these findings suggest that ABT263 can induce apoptosis in senescent BM-MSCs obtained from 1,25(OH)_2_D-insufficient mice.

### 3.3 ABT263 intervention alleviates bone loss caused by 1,25(OH)_2_D insufficiency

**Figure 3:**
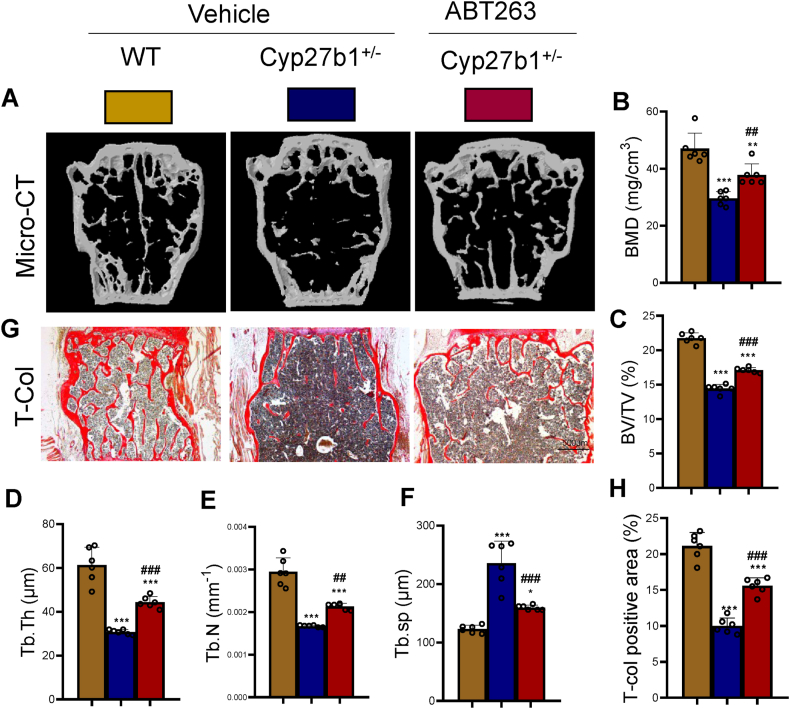
ABT263 intervention corrects bone loss caused by 1,25(OH)2D insufficiency Lumbar vertebrae of WT, Cyp27b1^+/−^, and ABT263 treated Cyp27b1^+/−^ (Cyp27b1^+/−^+ ABT263) mice. (A) Three-dimensional reconstruction by micro-CT. (B) Quantitative analysis of BMD (mg/cm^3^). (C) Quantitative analysis of BV/TV (%). (D) Quantitative analysis of Tb.Th (μm). (E) Quantitative analysis of Tb.N (mm^−1^). (F) Quantitative analysis of Tb.Sp (μm). (G) Representative micrographs of T-COL histological staining. (H) Quantitative analysis of total collagen (T-COL) staining-positive area (%). Values are mean ± S.E.M. of 6 determinations per group. ∗∗∗: P < 0.001, compared to WT mice. ##: P < 0.01; ###: P < 0.001, compared to Cyp27b1^+/−^ mice.

To evaluate the efficacy of ABT263 intervention in restoring bone loss caused by 1,25(OH)_2_D insufficiency, 11-month-old WT mice received a vehicle, while the littermate Cyp27b1^+/−^ mice were administered either a vehicle or ABT263. To evaluate the impact of ABT263 on bone health, we conducted a comparative analysis of vertebral phenotypes among wild-type (WT), Cyp27b1^+/−^, and ABT263-treated Cyp27b1^+/−^ mice. This analysis employed micro-computed tomography (micro-CT) imaging and histochemical staining for total collagen to discern any variations. The results of the study demonstrated significant improvements in bone mineral density, bone volume, trabecular number, trabecular thickness, and total collagen staining-positive area in the ABT263-treated Cyp27b1^+/−^ mice compared to the Cyp27b1^+/−^ mice (Fig. 3A–E, G & H). Additionally, there was a significant reduction in trabecular separation observed in the ABT263-treated mice (Fig. 3F). These findings provide in vivo evidence that ABT263 intervention effectively increases bone density, bone volume, trabecular number, and collagen synthesis, thereby rectifying the bone loss resulting from 1,25(OH)_2_D insufficiency.

### 3.4 ABT263 intervention alleviates decreased bone formation and increased bone resorption caused by 1,25(OH)_2_D insufficiency

**Figure 4:**
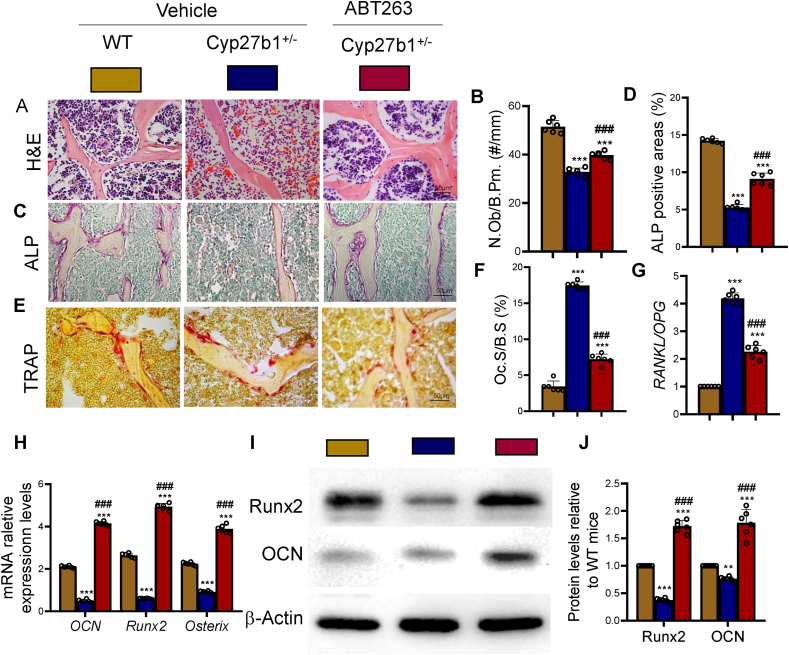
ABT263 intervention corrects decreased bone formation and increased bone resorption caused by 1,25(OH)_2_D insufficiency Slices of lumbar vertebrae from WT, Cyp27b1^+/−^, and Cyp27b1^+/−^+ ABT263 mice. (A) Representative micrographs of H&E staining. (B) Quantitative analysis of osteoblast numbers per unit bone tissue area (N.Ob/B.Pm., #/mm). (C) Representative micrographs of ALP histochemical staining. (D) Quantitative analysis of ALP-positive area (%). (E) Representative micrographs of TRAP histochemical staining. (F) Osteoclastic surface relative to bone surface (Oc.S/B.S, %). (G) Real-time RT–PCR of tissue extracts of lumbar vertebrae for expression of RANKL and OPG. Messenger RNA expression assessed by real-time RT-PCR is calculated as a ratio relative to Gapdh, and expressed as ratio of *RANKL*/*OPG* mRNA relative levels to WT mice. (H) Relative expression levels of *OCN*, *Runx2*, and *Osterix* mRNA. (I) Western blot results of OCN, Runx2, and Osterix proteins in thoracic vertebrae tissues. (J) Relative expression levels of OCN and Runx2 proteins. Values are mean ± S.E.M. of 6 determinations per group. ∗∗∗: P < 0.001, compared to WT mice. ##: P < 0.01; ###: P < 0.001, compared to Cyp27b1^+/−^ mice.

To investigate whether the intervention of ABT263 could alleviate accelerated bone loss caused by 1,25(OH)_2_D insufficiency, we examined its effects on osteoblastic bone formation. Relevant indicators were assessed using H&E staining, ALP histochemical staining, and Western blot analysis. Our results demonstrated that ABT263-treated Cyp27b1^+/−^ mice exhibited significantly increased numbers of osteoblasts, ALP-positive area, mRNA expression levels of *osteocalcin* (*OCN*), *Runx2*, and *Osterix*, as well as protein expression levels of Runx2 and OCN in spinal bone tissues (Fig. 4A–D & H-J). These findings suggest that ABT263 intervention alleviated the diminished bone formation caused by 1,25(OH)_2_D insufficiency by enhancing the osteogenic capacity of osteoblasts in Cyp27b1^+/−^ mice.

Additionally, we investigated whether the administration of ABT263 could alleviate the increased bone resorption caused by 1,25(OH)_2_D insufficiency by examining its effects on osteoclastic bone resorption. The relevant indicators were evaluated through histochemical staining for TRAP and real-time RT-PCR. Our results revealed that ABT263-treated Cyp27b1^+/−^ mice exhibited significantly reduced percentages of TRAP-positive osteoclastic surface and *RANKL*/*OPG* mRNA ratio in their spinal bone tissues (Fig. 4E–G). These findings indicate that ABT263 intervention addressed the heightened bone resorption caused by 1,25(OH)_2_D insufficiency by reducing osteoclast bone resorption in Cyp27b1^+/−^ mice.

### 3.5 ABT263 intervention alleviates oxidative stress and DNA damage caused by 1,25(OH)_2_D insufficiency

**Figure 5:**
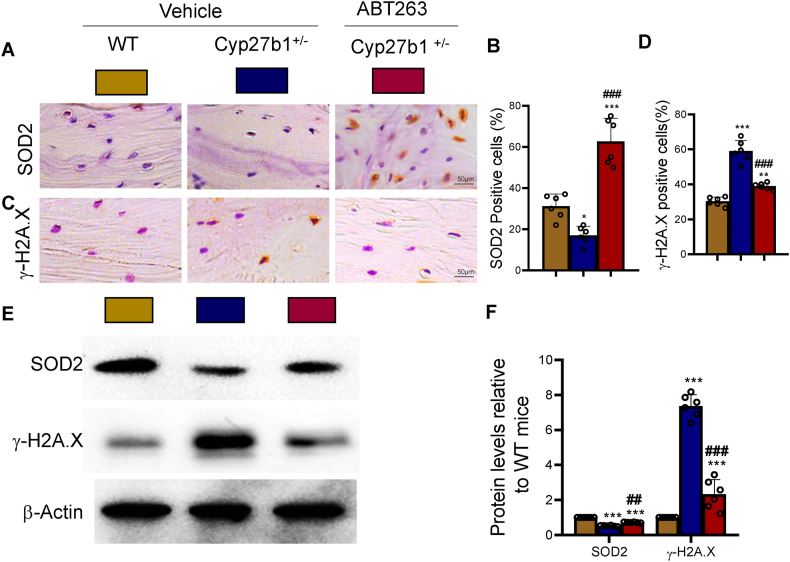
ABT263 intervention corrects oxidative stress and DNA damage caused by 1,25(OH)_2_D insufficiency Lumbar vertebrae of WT, Cyp27b1^+/−^, and Cyp27b1^+/−^+ ABT263 mice. (A & B) Representative micrographs of SOD2 immunohistochemical staining and the percentage of SOD2-positive cells. (C & D) Representative micrographs of γ-H2A.X immunohistochemical staining and the percentage of γ-H2A.X-positive cells. (E & F) Western blot results of SOD2 and γ-H2A.X proteins. Values are mean ± S.E.M. of 6 determinations per group. ∗∗∗: P < 0.001, compared to WT mice. ##: P < 0.01; ###: P < 0.001, compared to Cyp27b1^+/−^ mice.

To examine the potential of ABT263 intervention in mitigating accelerated bone loss caused by 1,25(OH)_2_D insufficiency by reducing oxidative stress and DNA damage, we performed a comprehensive comparative analysis. This analysis encompassed a range of oxidative stress and DNA damage-related biomarkers. The results revealed a significant decrease in the percentage of SOD2-positive bone cells and a reduction in the expression level of SOD2, an anti-oxidative protein, in bone tissues of Cyp27b1^+/−^ mice in comparison to WT mice. Conversely, these indicators showed a significant increase in ABT263-treated Cyp27b1^+/−^ mice (Fig. 5A, B, E & F). Furthermore, the percentage of γ-H2A.X-positive bone cells and the expression level of γ-H2A.X, a protein associated with DNA damage, displayed a marked increase in Cyp27b1^+/−^ mice as compared to WT mice. By contrast, these indicators demonstrated a noticeable decrease in ABT263-treated Cyp27b1^+/−^ mice (Fig. 5C–F). These findings suggest that ABT263 intervention can effectively counteract the bone loss triggered by 1,25(OH)_2_D insufficiency by inhibiting oxidative stress and DNA damage.

### 3.6 ABT263 intervention alleviates cellular senescence and SASP induced by 1,25(OH)_2_D insufficiency

**Figure 6:**
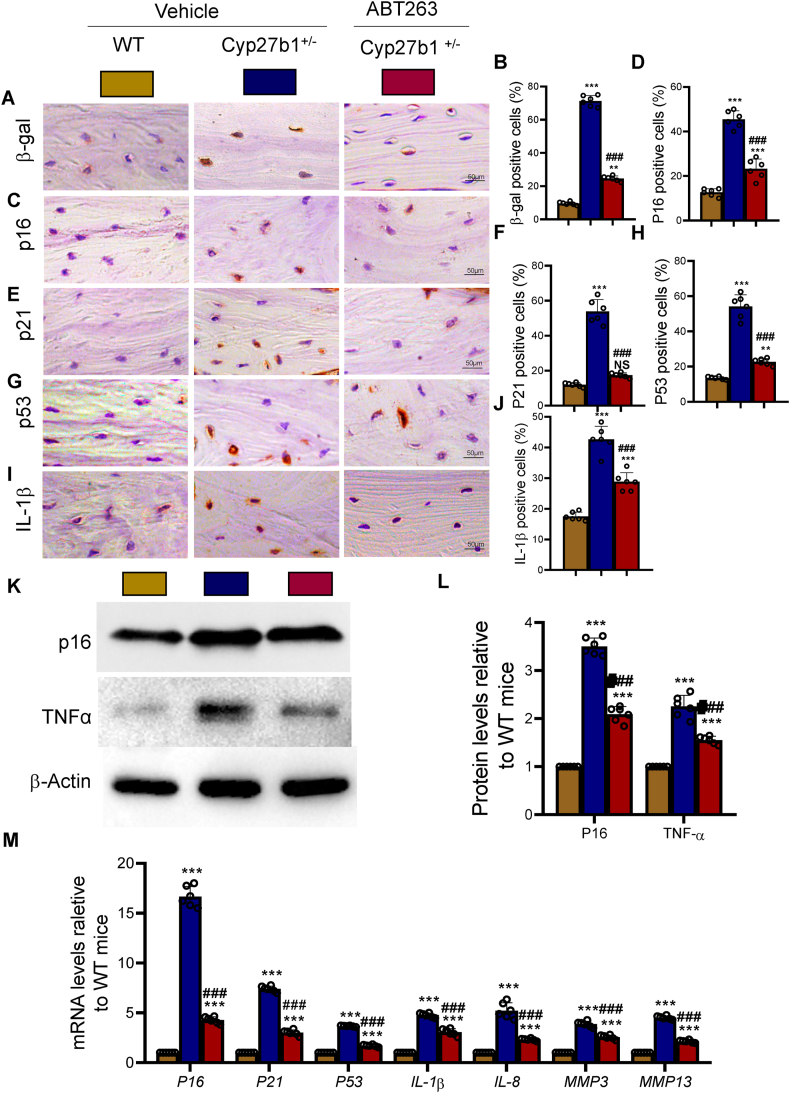
ABT263 intervention corrects bone cell senescence and SASP caused by 1,25(OH)_2_D insufficiency Slices of lumbar vertebrae from WT, Cyp27b1^+/−^, and Cyp27b1^+/−^+ ABT263 mice. (A & B) Representative micrographs and the percentage of β-gal-positive cells. (C & D) Representative micrographs and the percentage of p16-positive cells. (E & F) Representative micrographs and the percentage of p21-positive cells. (G & H) Representative micrographs and the percentage of p53-positive cells. (I & J) Representative micrographs and the percentage of IL-1β-positive cells. (K) Western blot results of p16 and TNFα proteins. (L) Relative expression levels of p16 and TNFα proteins. (M) Relative expression levels of senescence-related genes p16, p21, p53, and SASP-related genes *IL-1β*, *IL-8*, *MMP3*, and *MMP1*3 mRNA. Values are mean ± S.E.M. of 6 determinations per group. ∗: P < 0.05; ∗∗: P < 0.01; ∗∗∗: P < 0.001, compared to WT mice. ##: P < 0.01; ###: P < 0.001, compared to Cyp27b1^+/−^ mice.

To assess the impact of ABT263 intervention on mitigating the accelerated bone loss caused by 1,25(OH)_2_D insufficiency, we investigated the association between this intervention and alterations in cellular senescence and the senescence-associated secretory phenotype (SASP). This assessment was conducted through a comprehensive analysis comparing changes in senescence-related and SASP-related markers. Our findings revealed notable differences between the test groups. In comparison to the WT mice, the Cyp27b1^+/−^ mice displayed significantly elevated percentages of β-galactosidase-positive osteocytes, as well as heightened levels of p16, p21, p53, and IL-1β-positive osteocytes. Additionally, the expression of p16 and TNFα proteins in bone tissues, as well as the mRNA expression of the senescence-related genes (*p16*, *p21*, and *p53*) and SASP-related genes (*IL-1β*, *IL-8*, *MMP3*, and *MMP13*), were all substantially higher in the Cyp27b1^+/−^ mice (Fig. 6A–M). Conversely, the administration of ABT263 to the Cyp27b1^+/−^ mice resulted in significant reductions in the aforementioned indicators (Fig. 6A–M). These results indicate that ABT263 intervention effectively decreases cellular senescence and SASP, ultimately rectifying the accelerated bone loss caused by 1,25(OH)_2_D insufficiency.

### 3.7 ABT263 intervention restores impaired BM-MSC osteogenesis in response to 1,25(OH)_2_D insufficiency

**Figure 7:**
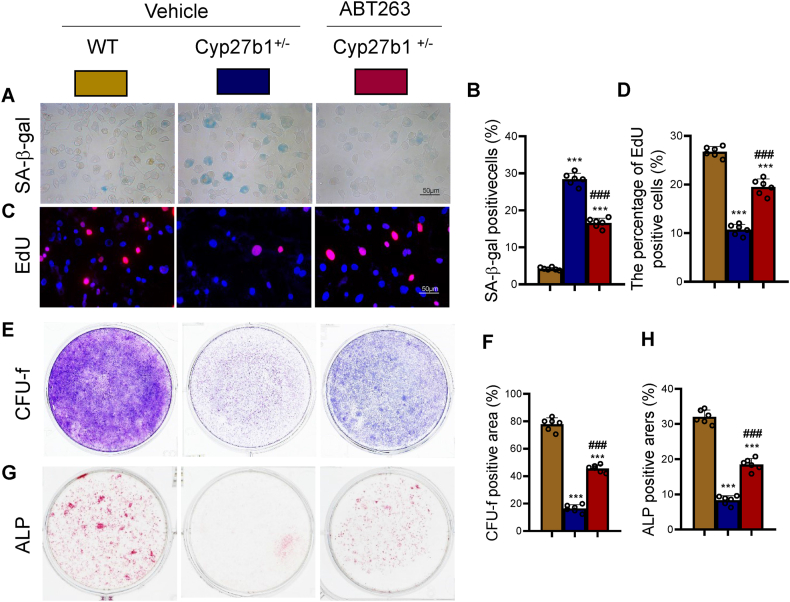
ABT263 intervention corrects BMSC osteogenesis impairments caused by 1,25(OH)_2_D insufficiency BM-MSCs from WT, Cyp27b1^+/−^, and Cyp27b1^+/−^+ ABT263 mice were treated for 1 month and were cultured *ex vivo*. (A) Representative micrographs of SA-β-gal staining. (B) Percentage of SA-β-gal-positive cells. (C) Representative micrographs of EdU incorporation. (D) Percentage of EdU-positive cells. (E) Representative image of methylene blue staining. (F) Quantification of CFU-f-positive area. (G) Representative micrographs of ALP cytochemical staining. (H) Quantification of ALP-positive area. Values are mean ± S.E.M. of 6 determinations per group. ∗∗∗: P < 0.001, compared to WT mice. ##: P < 0.01; ###: P < 0.001, compared to Cyp27b1^+/−^ mice. (For interpretation of the references to colour in this figure legend, the reader is referred to the Web version of this article.)

To elucidate the correlation between the enhanced osteoblast numbers via ABT263 treatment in Cyp27b1^+/−^ mice and the proliferation and osteogenic differentiation of BM-MSCs, *ex vivo* cultures of BM-MSCs were established from 12-month-old WT, Cyp27b1^+/−^, and ABT263-treated Cyp27b1^+/−^ mice. A comprehensive set of analyses was conducted, encompassing SA-β-gal cytochemical staining, ALP activity assessment, EdU incorporation, methylene blue staining, and ALP cytochemical staining. Changes in the size and number of colony-forming units-fibroblastic (CFU-f) and ALP-positive CFU-f were quantified using image analysis techniques. The results revealed a significant increase in the percentage of SA-β-gal-positive cells in BM-MSCs derived from Cyp27b1^+/−^ mice compared to WT mice. Conversely, this percentage decreased significantly in ABT263-treated Cyp27b1^+/−^ mice-derived BM-MSCs (Fig. 7A and B). Additionally, the number of EdU-positive cells, positive CFU-f area, and ALP-positive CFU-f area exhibited a significant decrease in BM-MSCs derived from Cyp27b1^+/−^ mice compared to WT mice. However, these parameters experienced a significant increase in ABT263-treated Cyp27b1^+/−^ mice-derived BM-MSCs compared to Cyp27b1^+/−^ mice (Fig. 7C–H). The present findings indicate that intervention with ABT263 can potentially rectify the impairments in osteogenesis of BM-MSCs caused by an insufficiency in 1,25(OH)_2_D. This result is achieved by reducing senescence in BM-MSCs, consequently promoting their proliferation and osteogenic differentiation.

## 4 Discussion

The present study investigates the potential of ABT263, a potent senolytic compound, as a therapeutic intervention for age-related bone loss and osteoporosis exacerbated by 1,25(OH)_2_D insufficiency. The findings provide compelling evidence that ABT263 can effectively ameliorate the accelerated bone loss observed in 1,25(OH)_2_D-insufficient mice by inducing apoptosis in senescent BM-MSCs and counteracting the detrimental effects of cellular senescence on bone homeostasis.

The accumulation of senescent cells in various tissues, including bone, is a hallmark of aging and has been implicated in the pathogenesis of age-related diseases. Senescent cells acquire a pro-inflammatory and catabolic secretory phenotype known as the senescence-associated secretory phenotype (SASP), which can disrupt tissue homeostasis and contribute to the development of various pathologies [14]. In the context of bone metabolism, the accumulation of senescent BM-MSCs has been linked to impaired osteoblast differentiation and bone formation, leading to age-related bone loss [17].

The present study demonstrates that ABT263 selectively induces apoptosis in senescent BM-MSCs derived from aged mice and 1,25(OH)_2_D-insufficient mice, while sparing non-senescent cells from young mice. The senolytic activity of ABT263 is mediated through the downregulation of the anti-apoptotic protein Bcl-2 and the upregulation of the pro-apoptotic proteins Bax and Caspase-3. These findings are consistent with previous studies that have reported the senolytic potential of ABT263 in various disease models [18,21].

Notably, the intervention with ABT263 effectively corrected the accelerated bone loss observed in 1,25(OH)_2_D-insufficient mice, as evidenced by significant improvements in bone mineral density, bone volume, trabecular number, trabecular thickness, and collagen synthesis. Additionally, ABT263 treatment reduced trabecular separation, a key indicator of bone microarchitecture deterioration [29]. These findings suggest that ABT263 can restore the impaired bone quality and strength associated with 1,25(OH)_2_D insufficiency, potentially reducing the risk of fractures and other bone-related complications. While ABT263 treatment significantly improved bone parameters in Cyp27b1^+/−^ mice, it is important to note that the restoration of bone loss was partial rather than complete. This observation can be attributed to several factors. First, vitamin D insufficiency affects bone metabolism through multiple mechanisms beyond cellular senescence, including altered calcium homeostasis and impaired mineralization [2,30]. ABT263, as a senolytic agent, primarily addresses senescence-related aspects of bone loss but may not fully correct other vitamin D-dependent processes. Second, the chronic nature of vitamin D insufficiency in our model likely led to cumulative effects that may not be entirely reversible through senolytic intervention alone [31]. Third, while ABT263 demonstrated significant senolytic activity, it may not eliminate all senescent cells in the bone microenvironment [15]. Fourth, the regenerative capacity of aged bone may be limited, even after senescent cell clearance, due to a reduced pool of functional osteoprogenitor cells or other age-related changes [32]. Finally, the duration of our treatment regimen or potential compensatory mechanisms triggered by senescent cell removal might have influenced the extent of bone mass restoration [13,33]. Future studies combining senolytic therapy with vitamin D supplementation or anabolic agents may lead to more complete restoration of bone mass in vitamin D-insufficient conditions [17].

Our findings of the protective effect of ABT-263 on bone in vitamin D insufficient mice appear to conflict with a recent report by Sharma et al. [34], which demonstrated that ABT-263 causes trabecular bone loss and impairs osteoprogenitor function in aged mice. This discrepancy likely stems from the different experimental contexts: vitamin D insufficiency-induced bone loss versus natural aging. The effects of ABT-263 may be highly dependent on the specific senescent cell populations present and the altered bone microenvironment in each condition [12,35]. Vitamin D insufficiency modifies the bone milieu in ways that could uniquely influence the response to senolytic treatment [36]. Furthermore, the balance between osteoblast and osteoclast activity may be differentially affected by ABT-263 in these distinct pathological states [37]. Our results suggest that the protective effect of ABT-263 may be specific to vitamin D insufficiency-induced bone loss, rather than a universal benefit to bone health. This underscores the complexity of cellular senescence in bone biology and the potential for context-dependent outcomes of senolytic therapies [15,33]. Future research should focus on characterizing senescent cell populations in various bone pathologies and elucidating the molecular mechanisms underlying these differential effects [38]. Such insights will be crucial for developing targeted senolytic approaches for specific bone disorders.

Furthermore, the study elucidates the underlying mechanisms by which ABT263 intervention corrects bone loss in 1,25(OH)_2_D-insufficient mice. ABT263 treatment effectively counteracted the diminished bone formation and enhanced bone resorption observed in these mice. Specifically, ABT263 intervention increased the number of osteoblasts, elevated alkaline phosphatase (ALP) activity, and upregulated the expression of osteogenic markers, including osteocalcin (OCN), Runx2, and Osterix. Concurrently, ABT263 treatment reduced osteoclastic bone resorption, as evidenced by decreased TRAP-positive osteoclastic surface and a lower *RANKL*/*OPG* mRNA ratio [39].

Interestingly, the study also revealed that ABT263 intervention counteracted the oxidative stress and DNA damage induced by 1,25(OH)_2_D insufficiency. ABT263 treatment increased the levels of the antioxidant enzyme superoxide dismutase 2 (SOD2) and reduced the expression of the DNA damage marker γ-H2A.X. These findings suggest that the senolytic activity of ABT263 may not only eliminate the detrimental effects of senescent cells but also mitigate the oxidative stress and genomic instability associated with cellular senescence [40].

Notably, ABT263 intervention alleviated cellular senescence and SASP in the bone tissues of 1,25(OH)_2_D-insufficient mice, as evidenced by decreased levels of senescence markers (p16, p21, and p53) and SASP-related inflammatory factors (IL-1β, MMP3, and MMP13). These findings highlight the potential of ABT263 to mitigate the pro-inflammatory and catabolic effects of senescent cells on bone homeostasis, further contributing to the correction of bone loss [41].

Importantly, the study demonstrates that ABT263 intervention can restore the impaired osteogenesis of BM-MSCs observed in 1,25(OH)_2_D insufficiency. *Ex vivo* analyses revealed that ABT263 treatment reduced senescence in BM-MSCs derived from 1,25(OH)_2_D-insufficient mice, promoting their proliferation and osteogenic differentiation. Specifically, ABT263 treatment increased the colony-forming unit-fibroblastic (CFU-f) and ALP-positive CFU-f areas, indicating enhanced proliferative capacity and osteogenic potential of BM-MSCs [28].

These findings suggest that the senolytic activity of ABT263 can restore the osteogenic capacity of BM-MSCs by eliminating the detrimental effects of senescent cells on their proliferation and differentiation potential. By rejuvenating the BMSC population, ABT263 intervention can potentially replenish the pool of osteoblast progenitors, thereby enhancing bone formation and counteracting the age-related decline in bone mass and quality [42].

ABT-263, a Bcl-2 family protein inhibitor studied for cancer treatment, has several significant safety concerns [43,44]. The primary safety concern for ABT-263 is thrombocytopenia, or low platelet count, which is consistently observed but generally considered tolerable. Its safety and efficacy profile varies depending on the expression of BCL-2 family proteins in target cells, suggesting potential variability in patient responses. Continuous dosing shows higher efficacy but may lead to stronger effects and potential toxicities compared to intermittent dosing. Some cancer cell lines exhibit resistance to navitoclax monotherapy, necessitating combination approaches that could introduce additional safety considerations. Furthermore, ABT-263 can enhance the effects of other chemotherapeutic agents, potentially increasing overall toxicity in combination regimens. While these concerns are noteworthy, further clinical data is needed to fully characterize its safety profile across different cancer types and treatment strategies.

Overall, this study provides a comprehensive evaluation of the therapeutic potential of ABT263 as a senolytic intervention for age-related bone loss and osteoporosis exacerbated by 1,25(OH)_2_D insufficiency. By selectively eliminating senescent BM-MSCs and counteracting their detrimental effects on bone homeostasis, ABT263 can effectively correct the accelerated bone loss observed in 1,25(OH)_2_D-insufficient mice. Furthermore, the study elucidates the underlying mechanisms by which ABT263 intervention restores bone formation, reduces bone resorption, mitigates oxidative stress and DNA damage, alleviates cellular senescence and SASP, and rejuvenates the osteogenic capacity of BM-MSCs.

These findings contribute to the growing body of evidence supporting the potential of senolytic therapies in the treatment of age-related diseases and open up new avenues for the development of novel therapeutic strategies targeting cellular senescence in the context of bone disorders and osteoporosis.

## Author contributions

D.M. and Z.D. conceived the project. C.Y. and W.Q. performed most of the experiments, analyzed, and compiled the data. Q.X. helped with experiments. D.M., Z.D. and D.G. participated in writing or editing the paper.

## Data availability statement

The datasets generated and analyzed during the current study are available from the corresponding author on reasonable request.

## Declaration of generative AI and AI-assisted technologies in the writing process

During the preparation of this work the authors used ChatGTP in order to improve language and readability. After using this tool, the authors reviewed and edited the content as needed and takes full responsibility for the content of the publication.

## Declaration of competing interest

The authors declare no conflicts of interest.
